# Soluble Prokaryotic Overexpression and Purification of Human GM-CSF Using the Protein Disulfide Isomerase b′a′ Domain

**DOI:** 10.3390/ijms22105267

**Published:** 2021-05-17

**Authors:** Thi Kieu Oanh Nguyen, Thi Luong Vu, Minh Quan Nguyen, Huynh Kim Khanh Ta, Kyoung Sun Park, Soo Hyeon Kim, Chong Jai Kim, Yeon Jin Jang, Han Choe

**Affiliations:** 1 Department of Physiology, Bio-Medical Institute of Technology, University of Ulsan College of Medicine, Asan Medical Center, Seoul 05505, Korea; ngtkioanh@gmail.com (T.K.O.N.); luongvu218@gmail.com (T.L.V.); minhquannguyen130295@gmail.com (M.Q.N.); khanhta3103@gmail.com (H.K.K.T.); yjjang@amc.seoul.kr (Y.J.J.); 2 Wide River Institute of Immunology, Seoul National University College of Medicine, Seoul 25159, Korea; kspark73@snu.ac.kr (K.S.P.); ksh5230@snu.ac.kr (S.H.K.); 3 Department of Pathology, Asan-Minnesota Institute for Innovating Transplantation, University of Ulsan College of Medicine, Asan Medical Center, Seoul 05505, Korea; ckim@amc.seoul.kr

**Keywords:** hGM-CSF, MBP, PDI, ClearColi

## Abstract

Granulocyte-macrophage colony-stimulating factor (GM-CSF) is a member of the colony-stimulating factor (CSF) family, which functions to enhance the proliferation and differentiation of hematopoietic stem cells and other hematopoietic lineages such as neutrophils, dendritic cells, or macrophages. These proteins have thus generated considerable interest in clinical therapy research. A current obstacle to the prokaryotic production of human GM-CSF (hGM-CSF) is its low solubility when overexpressed and subsequent complex refolding processes. In our present study, the solubility of hGM-CSF was examined when combined with three N-terminal fusion tags in five *E. coli* strains at three different expression temperatures. In the five *E. coli* strains BL21 (DE3), ClearColi BL21 (DE3), LOBSTR, SHuffle T7 and Origami2 (DE3), the hexahistidine-tagged hGM-CSF showed the best expression but was insoluble in all cases at each examined temperature. Tagging with the maltose-binding protein (MBP) and the b′a′ domain of protein disulfide isomerase (PDIb′a′) greatly improved the soluble overexpression of hGM-CSF at 30 °C and 18 °C. The solubility was not improved using the Origami2 (DE3) and SHuffle T7 strains that have been engineered for disulfide bond formation. Two conventional chromatographic steps were used to purify hGM-CSF from the overexpressed PDIb′a′-hGM-CSF produced in ClearColi BL21 (DE3). In the experiment, 0.65 mg of hGM-CSF was isolated from a 0.5 L flask culture of these *E. coli* and showed a 98% purity by SDS-PAGE analysis and silver staining. The bioactivity of this purified hGM-CSF was measured at an EC_50_ of 16.4 ± 2 pM by a CCK8 assay in TF-1 human erythroleukemia cells.

## 1. Introduction

Human granulocyte-macrophage colony-stimulating factor (hGM-CSF), also known as colony-stimulating factor 2 (CSF-2), enhances the growth and differentiation of hematopoietic progenitor cells to various lineages, including granulocytes, macrophages, eosinophils, megakaryocyte, and red blood cells [1], although it does not play a major role in steady-state myelopoiesis [2]. This protein also has a paracrine role in tissue inflammation, such as rheumatoid arthritis or multiple sclerosis [3]. Since this factor can also mobilize cells from the bone marrow into the blood, recombinant hGM-CSF has been used to treat neutropenia in cancer patients after radiotherapy or chemotherapy. Interestingly, hGM-CSF induces long-lasting, specific anti-tumor effects in mice [4]. However, subsequent clinical trials did not find any efficacy in human subjects [5]. The advance of immune-checkpoint cancer therapy has renewed interest in hGM-CSF cancer therapy in a combination with the immune-checkpoint antibody and other anti-cancer antibodies [6]. In addition to the clinical applications, hGM-CSF is used in the induction of hematopoietic cells from pluripotent stem cells. hGM-CSF also promotes the neuronal differentiation of adult neural stem cells [7]. Because of these clinical and non-clinical demands for hGM-CSF, efficient production of bioactive hGM-CSF has been pursued.

hGM-CSF is a 127-residue monomer with six glycosylation sites [8] and two types are available for clinical use. One is sargramostim, a glycosylated hGM-CSF from *Saccharomyces cerevisiae* [9], and the other is molgramostim, a non-glycosylated hGM-CSF from *E. coli* [10]. The glycosylation of hGM-CSF interferes with its own function [11] by decreasing its affinity to the receptor, GM-CSFR [12]. hGM-CSF has four cysteine residues that form two intramolecular disulfide bonds. The cytoplasm of *Escherichia coli* (*E. coli*) is a reducing environment however so that expressed recombinant hGM-CSF cannot be properly folded, and it thus driven into the aggregated structures known as inclusion bodies [13,14,15,16]. These aggregated proteins require cumbersome solubilization and refolding processes. Consequently, a number of different methodologies have been explored to achieve the soluble expression of hGM-CSF in *E. coli* including periplasmic expression [17], different *E. coli* strains [18], several additives and chaperone co-expression [19], thioredoxin-fusion [20], and intein-fusion [21].

In our present study, we tested additional approaches to addressing the solubilization and refolding problems with hGM-CSF by screening various combinations of holdase tags, *E. coli* strains, and temperatures. We also established a simple purification protocol for solubly overexpressed hGM-CSF fused to the protein disulfide isomerase (PDI) b′a′ domain. The hGM-CSF produced with this method showed a robust stimulatory effect on the proliferation of TF-1 cells.

## 2. Results

### 2.1. Design and Construction of Plasmids

**Figure 1:**
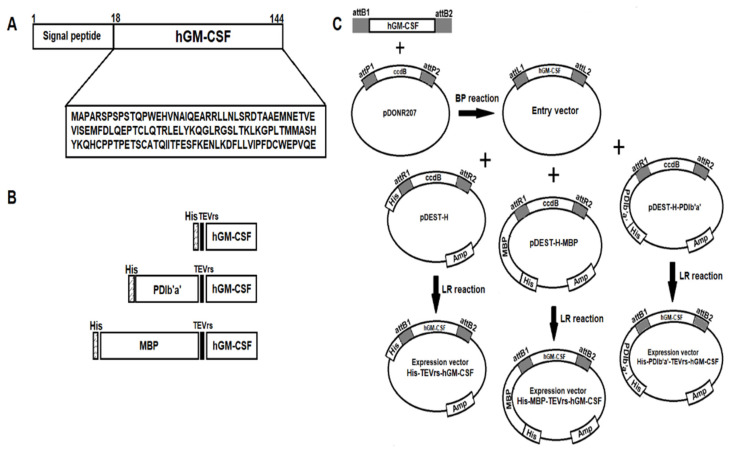
Construct of the hGM-CSF expression vectors. (**A**) Primary structure of hGM-CSF (amino acids 18-144). (**B**) Structure diagram of the His-hGM-CSF, PDIb′a′-hGM-CSF and MBP-hGM-CSF constructs. (**C**) Schematic of the Gateway cloning strategy to generate hGM-CSF expression vectors.

The core hGM-CSF sequence, i.e., without the signal sequence, was codon-optimized for expression in *E. coli* and then chemically synthesized (Figure 1). The TEV protease cleavage site (ENLYFQ/G) was next placed upstream of the hGM-CSF sequence and the resulting insert was cloned into the pDONR207 vector via a BP reaction to create the hGM-CSF entry vector. The hGM-CSF gene was then transferred from this entry vector into different plasmids harboring different tag proteins (His-tag, MBP tag, or PDIb′a′ tag) using the LR reaction to obtain the expression constructs His-TEV-hGM-CSF, MBP-TEV-hGM-CSF, and PDIb′a′-TEV-hGM-CSF. All expression vectors were verified by sequencing analysis prior to use.

### 2.2. Expression and Solubility of the Recombinant Tagged hGM-CSF Proteins

**Figure 2:**
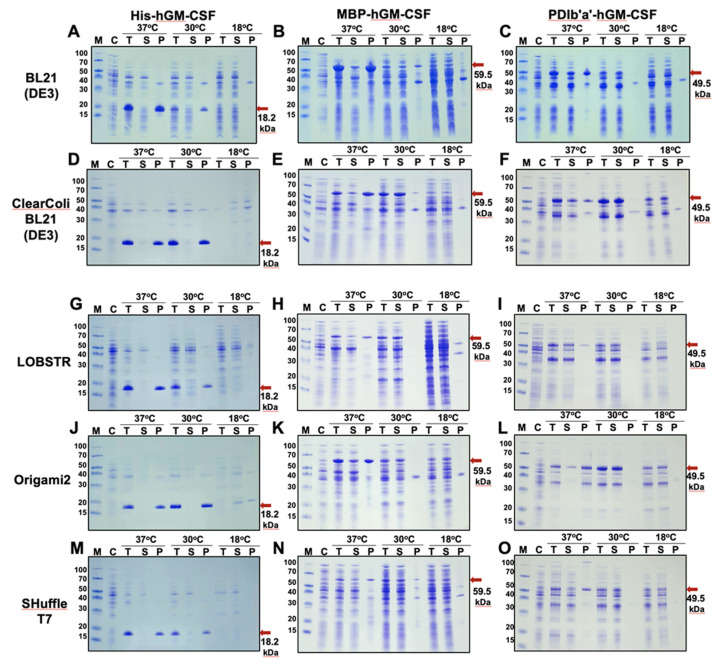
Expression and solubility analysis of fusion proteins produced in *E. coli*. Expression of the proteins was induced by the addition of 0.5 mM IPTG to the bacterial cultures at 18 °C, 30 °C, or 37 °C using different *E. coli* strains. Expression results are shown for the BL21 (DE3) strain (**A**,**C**), ClearColi BL21 (DE3) strain (**D**,**F**), LOBSTR strain (**G**,**I**), Origami2 (DE3) strain (**J**,**L**), and SHuffle T7 strain (**M**,**O**). The bacterial expression plasmids used were His-hGM-CSF (**A**,**D**,**G**,**J**,**M**), MBP-hGM-CSF (**B**,**E**,**H**,**K**,**N**), and PDIb′a′-hGM-CSF (**C**,**F**,**I**,**L**,**O**). M, protein molecular weight markers; C, total protein extract of *E. coli* prior to IPTG induction; T, total protein lysate after IPTG induction; S, supernatant portion of the total lysate after bacterial lysis; P, pellet portion of the total lysate after bacterial lysis. The red arrows indicate the His-hGM-CSF (18.2 kDa), MBP-hGM-CSF (59.5 kDa), and PDIb′a′-hGM-CSF (49.5 kDa) fusion protein products.

The His-TEV-hGM-CSF, MBP-TEV-hGM-CSF and PDIb′a′-TEV-hGM-CSF constructs were transformed into five different *E. coli* strains and recombinant protein production was induced by the addition of IPTG to the growth cultures at three different temperatures. The whole bacterial lysate, supernatant, and pellet portions were each subjected to SDS-PAGE (Figure 2), and then evaluated as the percentage of fusion protein compared to the total amount of total protein using Gel Analyzer software (Table 1). The fusion His-hGM-CSF proteins were overexpressed at 37 °C and 30 °C, mostly in insoluble form, in all of the tested *E. coli* strains, but expression was barely detectable from any culture grown at 18 °C (Figure 2A,D,G,J,M; Table 1). When using the MBP or PDIb′a′ tags in different *E. coli* strains, the resulting fusion proteins showed different expression levels in both soluble and insoluble forms (Table 1). Fusion proteins with the MBP tag produced at 37 °C in the Origami2 (DE3) strain (Figure 2K, Table 1) showed the expression (25.5%) with the highest solubility of 33.3%. The expression level results from the other *E. coli* strains were 22–30%, with most of the overexpressed proteins being in an insoluble form (Figure 2B,E,H,N; Table 1). At 30 °C in the in ClearColi BL21(DE3) strain, the MBP-hGM-CSF protein showed an expression level of 33.1% and solubility of 63.5%, which compared favorably with the production in other strains that showed expression levels of 14–20%, mostly consisting of fusion proteins in soluble forms. At 18 °C, MBP-hGM-CSF had a low expression level that was not sufficient for large scale protein purification. With regard to the PDIb′a′ tagged fusion proteins, the ClearColi BL21(DE3) (Figure 2F) and Origami2 (DE3) (Figure 2L) strains had a higher induced overexpression than the other strains at 37 °C and 30 °C (Table 1). At 18 °C, the ClearColi BL21(DE3), Origami2 (DE3), and SHuffle T7 strains (Figure 2F,L,O) showed expression levels of around 15% (Table 1), while a small protein expression could be detected in the LOBSTR strain. Overall, our results indicated that at a 30 °C incubation temperature and induction with 0.5 mM IPTG, the MBP-hGM-CSF and PDIb′a′-hGM-CSF products were mostly overexpressed in soluble forms in the ClearColi BL21 (DE3) and Origami2 (DE3) *E. coli* strains.

### 2.3. Growth Rates

**Figure 3:**
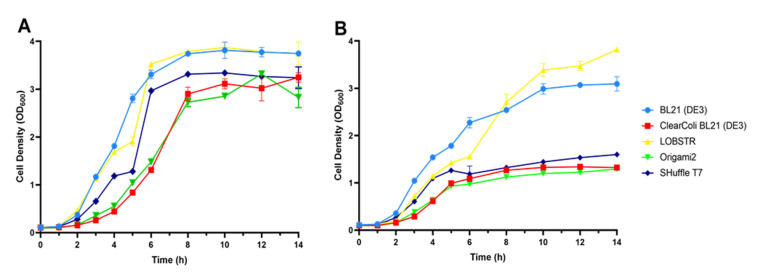
Growth curves of the indicated *E. coli* strains with and without induction. (**A**) *E. coli* strains transformed with the PDIb′a′-hGM-CSF plasmid were cultured at 30 °C without induction. (**B**) IPTG was added to induce the expression of PDIb′a′-hGM-CSF at 30 °C.

The growth rate of the host bacteria is another important factor for the overall production level of recombinant proteins. This was measured in our current analyses in a standard manner using optical density readings at a 600 nm wavelength (OD_600_; Figure 3). Without IPTG induction, all of the tested *E. Coli* strains reached a maximum OD_600_ at 8 h. The BL21 (DE3) and Lobster strains grew fastest, the SHuffle strain was in the middle, and the ClearColi BL21 (DE3) and Origami2 (DE3) strains showed the slowest growth rates (Figure 3A). In the second part of this experiment, protein expression was induced with IPTG when the OD_600_ reached 0.6–0.8. Compared to the control cultures, the growth of all of the strains was then reduced (Figure 3B). However, the order of the strains in terms of these rates was preserved. Another interesting observation was that the maximum OD_600_ was reduced significantly in the ClearColi BL21 (DE3), SHuffle, and Origami2 (DE3) strains.

### 2.4. Purification of Recombinant hGM-CSF

**Figure 4:**
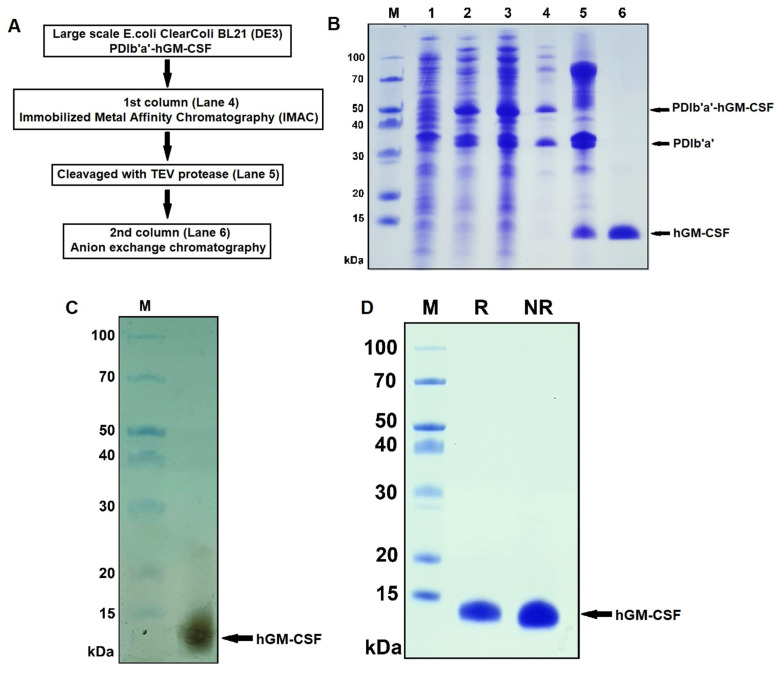
Purification of hGM-CSF from the PDIb’a’-hGM-CSF product harvested from *E. coli.* (**A**) Flow chart of the hGM-CSF purification steps. (**B**) hGM-CSF was purified by IMAC and anion-exchange chromatography from PDIb′a′-hGM-CSF produced in ClearColi BL21 (DE3) cultures. M, protein molecular weight markers. Lane 1, total lysate proteins prior to IPTG induction as a negative control; lane 2, total proteins induced by IPTG after cell sonication; lane 3, soluble proteins obtained after cell sonication of the total cell proteins; lane 4, the PDIb′a′-hGM-CSF fusion protein (49.5 kDa) purified with an IMAC column; lane 5, the result of TEV protease cleavage (28.6 kDa) yielding the PDIb′a′ tag (34.9 kDa) and hGM-CSF (14.6 kDa); lane 6, hGM-CSF (14.6 kDa) purified using ion-exchange chromatography. (**C**) Silver staining of hGM-CSF under reducing conditions. M, protein molecular weight markers. (**D**) Purified hGM-CSF protein under reducing and non-reducing conditions. M, protein molecular weight markers; R, reducing; NR, non-reducing.

Based on our soluble overexpression test results of the recombinant hGM-CSF in *E. Coli*, we chose the PDIb′a′-hGM-CSF from the ClearColi BL21 (DE3) strain, inoculated at 30 °C with IPTG induction. The final hGM-CSF preparation was purified using a 2-step chromatography approach (immobilized metal affinity chromatography (IMAC) and ion exchange chromatography (IEC)) with TEV protease cleavage after the IMAC step (Figure 4A). Prior to purification, the large-scale expression of fusion protein PDIb′a′-hGM-CSF was tested by SDS-PAGE, which confirmed that the fusion protein (Figure 4B, Lane 2) was overexpressed when compared with the non-IPTG induction culture (Figure 4B, lane 3). Because all our tags contain poly His at their N-termini, the PDIb′a′-hGM-CSF product could bind to Ni-resin column, and be effectively isolated with the elution buffer, and the protein fractions were then analyzed by SDS-PAGE (Figure 4B, Lane 4).

For recombinant hGM-CSF biochemical studies, the fusion tag in our PDIb′a′-hGM-CSF product was eliminated by digestion with the tobacco etch virus (TEV) protease, as the cleavage site for this enzyme had been placed downstream of the PDIb′a′-tag. The dialyzed fractions of this fusion protein obtained with IMAC were cleaved with the TEV protease, and then verified using SDS-PAGE (Figure 4B, Lane 5).

**Figure 5:**
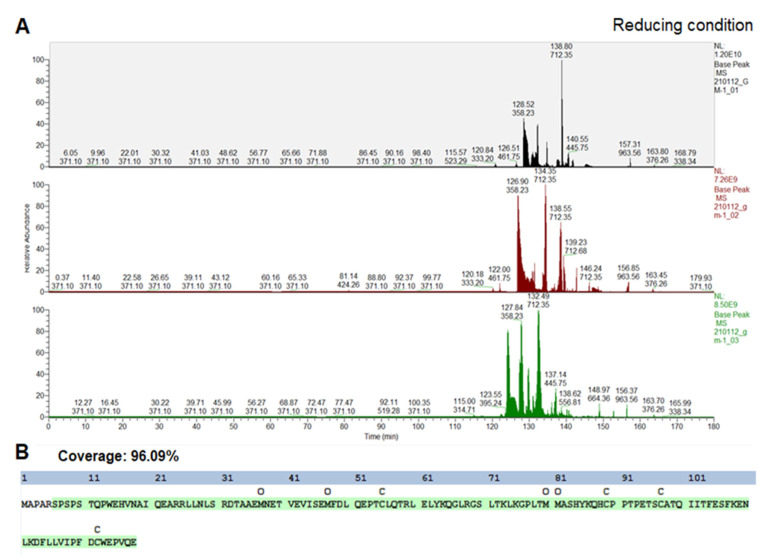
LC-MS/MS analysis of the purified hGM-CSF. (**A**) LC-MS/MS sequencing of peptides for protein identification was performed using trypsin for sample digestion and under reducing conditions. The arrows indicate peaks matching corresponding peptide fragments. (**B**) The covered sequence is presented using a color-coded display based on percolator confidence scores (green, high confidence, *p*-value < 0.01). Coverage was 96.09%.

Both the target protein and PDIb′a′-tag were next purified by anion exchange chromatography based on the isoelectric point of hGM-CSF. The purity of the eluted hGM-CSF (14.6 kDa) (Figure 4B, Lane 6) was found to be 97.5% (Table 2) when a silver stained SDS-PAGE gel (Figure 4C) was analyzed using Gel Analyzer software. Under reducing SDS-PAGE conditions, the hGM-CSF product appeared as a single band with a lower mobility than under non-reducing conditions (Figure 4D). Typically, we found that a 0.5 L culture of E. coli expressing PDIb′a′-hGM-CSF could yield 0.65 mg of hGM-CSF with a purity 97.5%, which represented 13.5% of the total protein in the lysate (Table 2). To verify the identity of the purified hGM-CSF protein, LC-MS/MS analysis was performed (Figure 5). Multiple fragments were detectable with a coverage of 96.09%.

### 2.5. Biological Activity of the Purified hGM-CSF

**Figure 6:**
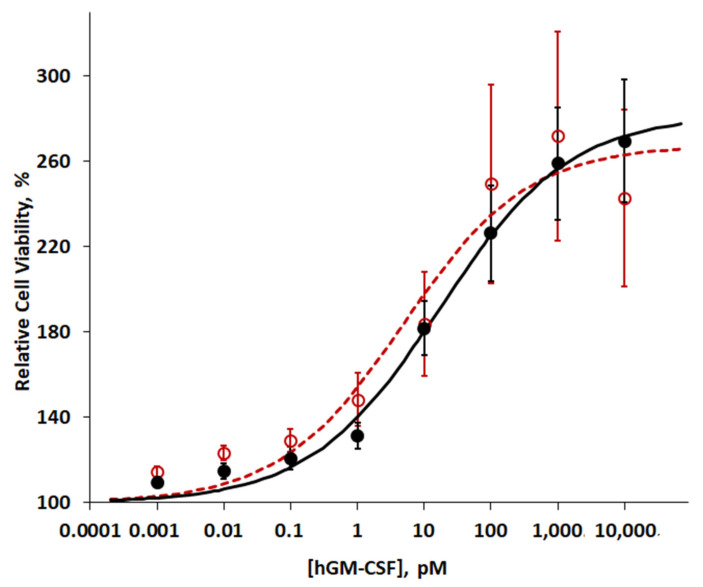
Biological activity of the purified hGM-CSF. The biological activities of purified hGM-CSF (black solid circle with solid line) and commercial hGM-CSF (red open circle with broken line) were tested by their ability to promote the proliferation of TF-1 cells. Cell proliferation was measured after 48 h of treatment using a CCK8 assay. The EC_50_ is defined as the effective concentration of the growth factor at which cell proliferation reaches 50% maximum growth. The EC_50_ and the Hill coefficient for the purified hGM-CSF on TF-1 cells were calculated as 16.4 ± 2 pM and 0.49 ± 0.08 (*n* = 3), respectively. Some of the standard error bars are hidden by the data symbols.

The biological effects of the purified hGM-CSF proteins were tested on the TF-1 human leukemia cell line using the CCK8 assay, which measures cell viability. Exposure of these cells to our purified hGM-CSF stimulated their growth in a dose-dependent manner similar to that of the commercial hGM-CSF (Figure 6). The Hill equation was used to model the sigmoid shape of the dose-response curve. The EC_50_ and Hill coefficient of the purified hGM-CSF were 16.4 ± 2 pM and 0.49 ± 0.08 (*n* = 3), respectively. For the commercial hGM-CSF, the EC_50_ and Hill coefficient were 10.3 ± 3.4 pM and 0.63 ± 0.22 (*n* = 3), respectively.

## 3. Discussion

### 3.1. Expression and Solubility

Many heterologously expressed proteins, especially those that contain disulfide bonds, tend to misfold in the cytoplasm of *E. coli* and aggregate to form inclusion bodies. Recombinant GM-CSF also accumulates in this insoluble fraction when expressed in *E. coli* and requires manual solubilization and refolding [13,14,15,16]. In our current study, three different parameters, i.e., the *E. coli* strain, expression temperature, and protein tag, were varied to determine the best protocol for obtaining a soluble and substantial hGM-CSF preparation from a bacterial expression system. Among these three variables, the tag was found to be the most influential (Figure 2 and Table 1). In our previous studies, we tested seven or eight different tags to try and enhance the solubility of their passenger proteins [22,23,24,25,26,27,28,29,30]. Only three tags, i.e., His, MBP, and PDIb′a′, were tested in our current analysis however as MBP and PDIb′a′ have consistently shown good results and the very well-established His tag was employed as a control. Indeed, both the MBP and PDIb′a′ tags made the GM-CSF protein highly soluble when expressed in *E. Coli* (Figure 2 and Table 1).

The mechanisms underlying the positive solubilization effects of the MBP and PDIb′a′ tags remain to be determined. MBP is a well-known enhancer of passenger protein solubility [31,32] and has been suggested to act as a holdase, whereby it goes through a transient physical interaction between a folded MBP moiety and an incompletely-folded passenger protein [32]. A number of hydrophobic patches on its surfaces and its open conformation were also shown to be important for this activity of MBP [33]. PDI is a modular polypeptide consisting of four homologous domains, a, b, b′, and a′, plus a C-terminal extension, c [34]. PDIb′a′, which comprises two flexibly-linked domains, b′ and a′, is the minimum region needed for enzyme activity [35]. It has been demonstrated also that PDIb′a′ contains hydrophobic patches [36]. A single domain of PDIb′a′ alone, i.e., either b′ or a′, does not show enhanced solubility effects on passenger proteins (data not shown).

The hGM-CSF protein product has two intramolecular disulfide bonds and the naturally reducing environment in the cytoplasm of *E. coli* would thus make it difficult for it to properly fold. The *E. coli* strains SHuffle T7 and Origami2 (DE3) were developed to promote better cytoplasmic disulfide bond formation by engineering them to express trxB, gor, and ahpC* genes. In addition to these genes, SHuffle T7 expresses DsbC, which further alters the redox state of the cytoplasm [37]. In our previous studies, we found that the use of the SHuffle T7 or Origami2 (DE3) strains increased the solubility of the expressed passenger proteins [30,38]. Not surprisingly, this effect was more dramatic for albumin than for oncostatin M, which contain 17 and 2 disulfide bonds, respectively. In our present study, however, neither of these strains improved the solubility nor increased the expression level of hGM-CSF (Table 1), suggesting that the lack of disulfide bond formation is not the principal reason for the insolubility of this protein when expressed in bacteria.

As indicated in Table 1, lowering the induction temperature enhanced hGM-CSF solubility in our current experiments, consistent with the findings of many previous reports [39]. If the folding of a protein is mediated by an enzyme, a higher number of soluble proteins will be produced at a higher temperature if this increases the activity levels of that enzyme. Our current results suggested however that the folding of hGM-CSF in *E. coli* is not mediated enzymatically. Another possibility in this regard is that a higher temperature increases the production of the protein but with a more limited quality control, leading to the generation of more misfolded protein. However, low temperature cultivation can decrease the growth rate of *E. coli* [40] and consequently the protein expression level (Table 1).

### 3.2. Purification

The lower mobility of hGM-CSF in a reducing SDS-PAGE gel suggests that the purified protein contains disulfide bonds that make it compact. The single band that was obtained under both the reducing and non-reducing condition also suggested that the purified GM-CSF was a monomer. We successfully obtained 0.65 mg of hGM-CSF with a 97.5% purity from a 0.5 L flask culture (Table 2), which was a final yield of 1.3 mg/L. This level needs to be improved considerably considering that other studies using flask culture method have reported yields ranging from 7 to 20 mg/L for hGM-CSF production [15,19,21]. A fermentation process using a bioreactor increased the yield to 90 mg/L [16]. Another study has found that the yeast, *Pichia pastoris* can produce a yield of up to 760 mg/L of mouse GM-CSF when the culture is batch-fed [41]. The lower yields we obtained in this study are partially due to the low cell density of the ClearColi strain when this protein was expressed (Figure 3B).

Endotoxin has been always a problem when producing human proteins in Gram-negative bacteria such as *E. coli*. Several methods have been applied to ameliorate this such ultrafiltration, activated carbon, surfactants, anion-exchange chromatography, histamine- and histidine-immobilized Sepharose, and polymyxin B-immobilized Sepharose [42,43,44,45,46,47,48]. In our present experiments, we used the genetically modified *E. coli* strain, ClearColi BL21 (DE3), as it produces a modified and non-immunogenic LPS [49]. This eliminates the need for an endotoxin-removal step. More importantly it also removes the danger of subsequent endotoxin toxicity in a clinical setting. From our initial results with the improved production of soluble hGM-CSF in *E. Coli,* we anticipate that more optimal protocols can be developed in the future to give a higher yield and better biological activity.

### 3.3. Biological Activity

The purified hGM-CSF proteins we obtained from *E. Coli* showed a robust stimulatory effect on the proliferation of TF-1 cells, a human leukemia cell line that responds to multiple cytokines, such as interleukin-3 and GM-CSF. The EC_50_ of this stimulation was 16.4 ± 2 pM, which was equivalent to 0.23 ± 0.03 ng/mL (Figure 6). This value is somewhat higher than the 10–80 pg/mL reported in previous studies [15,18]. Considering similar results of the commercial hGM-CSF (Figure 6), the value we obtained may have been the results of a systematic error with the biological assays and may also have been related to the stability of the purified protein after freeze-thawing. Improvements in both instances will be required in the future.

## 4. Materials and Methods

### 4.1. Materials

Ampicillin and 2-(N-morpholino)ethanesulfonic acid (MES monohydrate) were obtained from Duchefa Biochemie (Haarlem, Netherlands). Dithiothreitol (DTT) and 1-thio-β-d-galactopyranoside (IPTG) were acquired from Anaspec (Fremont, CA, USA). Tris-Cl and Coomassie brilliant blue R-250 were purchased from Amresco (Solon, OH, USA). Other chemicals such as sodium hydroxide, sodium chloride, and glycerol were acquired from Samchun Chemical (Pyeongtaek, Korea) and imidazole was purchased from Daejung Chemicals (Siheung, Korea). Protein Marker (EBM-1014) were sourced from Elpis (Seoul, Korea). Silver Staining kit Plus was obtained from Bio-Rad Laboratories (Hercules, CA, USA).

The BL21 (DE3) *E. coli* strain was obtained from Invitrogen (Carlsbad, CA, USA). ClearColi BL21 (DE3) *E. coli* strain was purchased from Lucigen (Middleton, WI, USA). The LOBSTR *E. coli* strain was modified from the BL21(DE3) strain and was obtained from Kerafast (Boston, MA, USA). The Origami2 (DE3) *E. coli* strain was purchased from Novagen (Darmstadt, Germany). The SHuffle T7 *E. coli* strain was obtained from New England Biolabs (Ipswich, MA, USA). The TF-1 cell line was obtained from ATCC (Manassas, VA, USA).

The cell disruptor JY99-IIDN was purchased from Ningbo Scientz Biotechnology (Guangdong, China) and the Smart R17 micro refrigerated centrifuge was sourced from Hanil Science Industrial (Gimpo, Korea). The polyethersulfone (PES) membrane (filter pore size, 0.45 µm) and syringe filter (pore size, 0.22 µm) were purchased from Hyundai Micron (Seoul, Korea). Acrodisc syringe filters were acquired from Pall Korea (Seoul, Korea). Dialysis membranes were purchased from Viskase (Darien, IL, USA) and Amicon Ultra was purchased from Merck Millipore (Billerica, MA, USA). The IMAC column, Anion exchange column, and ÄKTA Start system were purchased from GE Healthcare (Piscataway, NJ, USA).

Cell culture materials and assay kits were purchased from different suppliers, including RPMI-1640 growth medium powder from GIBCO (Carlsbad, CA, USA), fetal bovine serum (FBS) from Avantor (Radnor, PA, USA), recombinant human granulocyte-macrophage colony-stimulating factor (hGM-CSF) from Peprotech (Cranbury, NJ, USA), penicillin-streptomycin from Biological Industries (Kibbutz Beit-Haemek, Israel), and Cell Counting Kit-8 from Dojindo (Rockville, MD, USA).

### 4.2. Plasmid Construction

All bacterial expression vectors used in the current study were constructed using the BP and LR reactions of the Gateway cloning system. The lambda integrase and excisionase which were used in the BP and LR reactions were supplied by Elpis Biotech (Daejeon, Korea). The hGM-CSF gene region encoding amino acids 18–144 of the protein product was codon-optimized for *E. coli* expression and synthesized by Cosmogenetech (Seoul, Korea). The tobacco etch virus recognition site (TEVrs: ENLYFQ/G) sequence was inserted at the N-terminus of the hGM-CSF (18–144) gene. The entry vector pENTRY-hGM-CSF was created via a BP reaction through the homogenous recombination of the attB and attP sites on the synthesized gene with the pDONR207 vector, in accordance with the manufacturer’s instructions. This entry vector was then recombined with other destination vectors (pDEST-H, pDEST-H-G-PDIb′a′, and pDEST-MBP-H) to generate expression constructs with different tag proteins. These expression vectors were validated by sequencing analysis prior to use (Cosmogenetech, Seoul, Korea).

### 4.3. Expression and Solubility of Fusion Proteins

The recombinant plasmids His-hGM-CSF, PDIb′a′-hGM-CSF, and MBP-hGM-CSF were transformed into the *E. coli* strains BL21 (DE3), ClearColi BL21(DE3), LOBSTR, SHuffle T7, and Origami2 (DE3) using the heat shock method to test their expression efficiency of recombinant hGM-CSF. To screen for colonies expressing high levels of protein, transformed *E. coli* were grown on LB agar plates containing 100 μg/mL ampicillin and selected colonies were inoculated in LB media also containing 100 μg/mL ampicillin and cultured overnight at 37 °C, with shaking at 250 rpm. The overnight suspension was inoculated the following day at a 1:100 ratio into 10 mL fresh LB medium supplemented with 100 μg/mL ampicillin and then cultured at 37 °C, with shaking at 250 rpm until the OD_600_ reached 0.6–0.8. Next, the *E. coli* suspension was separated into three different round-bottom tubes and induced with 0.5 mM IPTG. These tubes were cultured under distinct conditions including 37 °C for 4 h, 30 °C for 6 h, and 18 °C overnight with shaking at 200 rpm. The bacterial pellets were collected from 1 mL of each culture by centrifugation at 13,000 rpm for 5 min at 4 °C. These pellets were then lysed on ice using an ultrasonic cell disruptor with a 35% amplitude, and 5 sec ON/5 sec OFF pulse for 10 cycles. The lysed bacterial suspensions were then centrifuged at 13,000 rpm for 5 min at 4 °C to separate the soluble and insoluble fractions. The expression and solubility level of the hGM-CSF protein under each set of experiment conditions was evaluated using 10% tricine SDS-PAGE and Gel Analyzer software (http://www.gelanalyzer.com).

### 4.4. Purification of Recombinant hGM-CSF (18-144 aa) and Tag Removal

For purification of the recombinant hGM-CSF protein, a single bacterial colony was selected for seeding into a large-scale culture. The single colony was first inoculated in 10 mL of LB supplemented with 100 μg/mL ampicillin at 37 °C, with shaking at 180 rpm, overnight. This overnight *E. coli* cultured was inoculated the following day at a 1:100 ratio into 1 L of fresh LB medium containing 100 μg/mL ampicillin and cultured at 37 °C with shaking at 250 rpm until the OD_600_ was approximately 0.6–0.8. IPTG was then added to a final concentration of 0.5 mM and the culture was shifted to growth at 30 °C for a further 6 h. The bacteria were then harvested by centrifugation at 3500× *g* for 45 min at 4 °C. After centrifugation, the supernatant was removed, and the bacterial pellets were stored at −20 °C.

To extract the recombinant protein, the stored bacterial pellets were first resuspended in 50 mL of Tris buffer (50 mM Tris-HCl, 5% glycerol (*v*/*v*), pH 10.0 with 1× cocktail protease inhibitor) and disrupted by sonication in an ice-bath with an ultrasonic cell disruptor. The lysed cells were then centrifuged at 11,000× *g* for 25 min at 4 °C and the supernatant was filtered through a 0.45 μm pore membrane. The filtered supernatant was added to buffer at a final concentration of 500 mM NaCl and 25 mM imidazole and applied to an IMAC column (HiTrap FF 5 mL) which had been pre-equilibrated with a buffer containing 50 mM Tris-HCl, 500 mM NaCl, 20 mM imidazole 5% glycerol (*v*/*v*), and a pH of 8.0. After loading of the protein sample, the IMAC column was washed with at least 5 column volumes (CV) of the equilibration buffer to remove non-specifically bound proteins. To increase the purity of the protein, the column continued to be washed with 10 CV of 7% elution buffer (50 mM Tris-HCl, 500 mM NaCl, 1 M Imidazole, 5% glycerol (*v*/*v*), pH 8.0). The protein was eluted with 400 mM imidazole. The fractions containing the fusion protein were dialyzed in Tris buffer (50 mM Tris-HCl, pH 8.0) at a 1:10,000 ratio to remove the NaCl and imidazole. The fusion protein was then treated with TEV protease (Addgene: 8827) to remove the PDIb′a′ tag (at a 10:1 (*w*/*w*) ratio of the fusion protein and TEV protease) at 18 °C for 16–20 h.

The dialyzed sample was next exchanged for MES buffer, pH 6.5, and applied to a HiTrap Q HP 5 mL column which had been equilibrated with 50 mM MES, pH 6.5. After washing with 10 CV of 10% elution buffer (50 mM MES pH 6.5, 1 M NaCl), the hGM-CSF protein was eluted in 5 CVs of 20% elution buffer (corresponding to 200 mM NaCl) and then exchanged in PBS, pH 7.4, using a desalting column. The purified hGM-CSF was concentrated by centrifugation in a 3 K Amicon Ultra column at 3000× *g*, 4 °C, until it reached the desired volume. The fractions obtained at each purification step were analyzed using 10% Tricine SDS-PAGE. Protein concentrations were measured using BCA assay method in accordance with the manufacturer’s instructions.

### 4.5. SDS-PAGE and Silver Staining

Proteins were separated on 10% Tris-Tricine gels and visualized by staining with Coomassie Brilliant Blue R-250. The expression, solubility and purity of these products were quantified using Gel Analyzer software. Silver staining was performed using the Silver Staining kit Plus. Briefly, following electrophoresis, the polyacrylamide gel was placed in fixative solution (50% methanol, 10% acetic acid, 40% distilled water) for 20 min and then washed twice in distilled water for 10 min to increase the sensitivity and contrast of the staining. The staining and development steps required approximately 20-min incubations with subsequently mixed fresh solutions (prepared before use) of silver complex solution, reduction moderator solution, image development reagent and development accelerator solution. The stained gel was placed in 5% acetic acid and rinsed in high purity water to stop the reaction.

### 4.6. Analysis of SDS-PAGE Gels

Expression testing of the hGM-CSF fusion proteins and all protein sample testing during the purification steps was conducted using 10% Tricine SDS-PAGE. Briefly, the protein fractions were boiled for 10 min at 100 °C in 5× sample buffer (250 mM Tris-HCl, pH 6.8, 30% glycerol, 10% SDS, 0.01% bromophenol blue, 300 mM DTT) to denature the proteins. Under non-reducing conditions, the protein sample was applied in the 5X sample buffer without DTT. To detect protein bands, SDS PAGE gels were stained with Coomassie brilliant blue R-250 solution. The expression and solubility levels of the fusion proteins and the purity of the target proteins were analyzed using Gel Analyzer software and calculated using a Microsoft Excel spreadsheet using the equations:

Expression level = *F/T* (1)

Solubility = *S*/(*S + P)* (2)

where *F* is the amount of fusion protein; *T* is the total amount of cellular protein after induction with IPTG; *S* is the amount of fusion protein in the supernatant (S) and *P* is the amount of fusion protein in the pellet (P).

### 4.7. Identification of Purified hGM-CSF by LC-MS/MS

The purified hGM-CSF protein band in the SDS-PAGE gels was collected and analyzed by liquid chromatography with tandem mass spectrometry (LC-MS/MS) which can both accurately identify and quantify the substance in question. LC-MS/MS is a combination of mass spectrometry (MS), that provides a structural identity of individual components with high specificity and detection sensitivity, and high-performance liquid chromatography (HPLC) that can separate mixtures with multiple components. Briefly, the gel was washed and digested into peptides by trypsin, which were harvested and desalted if necessary. The protein was verified with GelDoc (Protein A280) and analyzed by LC-MS/MS. In the LC phase, the sample was separated using a PepMap RSLC 75 × 50 column containing solution A (0.1% formic acid, 2% Acetone in water), and solution B (0.1% formic acid in Acetonitrile) over 200 min with a flow rate 200–280 nL/min. Fractions were gradient-eluted from 5% solution B to 90% solution B in 180 min. The MS analysis was separated in two phases: MS1 with a resolution of 70,000 and maximum fill time of 50 msec in which the results were analyzed by data-dependent acquisition (DDA) methods; and MS2 with a resolution of 17,500 and maximum fill time of 150 msec, whereby the auto gain control for the peaks was 1 × 10^5^.

### 4.8. In Vitro Activity Assay

The in vitro biological activity of the purified hGM-CSF was measured by its ability to enhance the proliferation of the human erythrocytic cell line, TF-1. TF-1 cells were cultured in RPMI 1640 medium, 10% fetal bovine serum (FBS), 2 ng/mL hGM-CSF (Peprotech, 300-03), and 1% penicillin and streptomycin in a 37 °C, 5% CO_2_ incubator. The cells were washed with PBS to remove all cytokines, plated at a density of 7500 cells per well in a 96-well plate, and then maintained without hGM-CSF or FBS in the growth medium for 24 h before the activity test. The TF-1 cells were then treated with different concentrations of hGM-CSF from 0.01 pM to 10 nM (0.001, 0.01, 0.1, 1, 10, 100, 1000, and 10,000 pM) for 48 h at 37 °C in a humidified atmosphere of 5% CO_2_. The negative control was TF-1 cells treated with PBS and the blank control was the culture media containing the highest concentration of hGM-CSF without cells. All of these experiments were repeated three times. After a 48 h incubation, 10 μL CCK-8 reagent was added to each well and the plate was incubated for 4 h at 37 °C in the dark. The absorbance of the sample was measured at 450 nm with a microplate reader. The data were processed with Microsoft Excel software using the following Hill equation:

*Re* = *Bl* + (*Max* − *Bl*)/(1 + (*EC*_50_/*conc.*)*^Hs^*) (3)

where *Re* is the response of the cells, *Bl* is the baseline at the low concentration of exposure, *Max* is the maximum response, *conc.* is the concentration of the protein, and *Hs* is the Hill coefficient of stimulation.

### 4.9. Statistical Analysis

All data are presented as the mean ± standard error. Group means were compared using the Student’s *t*-test or one-way analysis of variance (ANOVA). *p* < 0.05 was considered significant.

## Figures and Tables

**Table 1:** Expression and solubility levels of His-hGM-CSF, MBP-hGM-CSF, and PDIb′a′-hGM-CSF.

		Expression Level (%)			Solubility (%)		
*E. Coli* strain	Tag	37 °C	30 °C	18 °C	37 °C	30 °C	18 °C
BL21 (DE3)	His	18 ± 1.9	17.3 ± 2.7	2.7 ± 0.8	22.7 ± 1.3	15.7 ± 1.7	80.2 ± 3
	MBP	22.1 ± 3.6	20.2 ± 4.9	7.3 ± 1	30.5 ± 5.1	42.7 ± 3.6	87.6 ± 2.4
	PDIb′a′	17.4 ± 1.4	14.1 ± 2.8	3.8 ± 1	26.7 ± 5.1	85 ± 3.6	77.7 ± 8.6
ClearColi BL21 (DE3)	His	66.3 ± 9.4	61.1 ± 5.6	9.3 ± 1.1	8.5 ± 2.4	7.5 ± 1.5	84.1 ± 5.3
	MBP	29.8 ± 2.9	33.1 ± 2.3	9.4 ± 1.7	25 ± 0.3	63.5 ± 14.6	84.8 ± 7.7
	PDIb′a′	24 ± 6.2	27.3 ± 6.6	13.3 ± 3.5	56 ± 2.1	94.6 ± 2.1	87.2 ± 6
LOBSTR	His	59.2 ± 2.9	38.9 ± 3.9	9.3 ± 0.4	10.5 ± 4.5	9.3 ± 0.7	68.4 ± 2.1
	MBP	22.4 ± 1.3	14.8 ± 3.3	6.8 ± 0.5	31 ± 1.1	68.2 13.4	86.5 ± 6.7
	PDIb′a′	17.2 ± 3.7	14.2 ± 2.6	3.7 ± 1.3	46.2 ± 2.1	89.7 ± 7.9	53.8 ± 11
Origami2 (DE3)	His	56.5 ± 5.8	50 ± 4.4	25.9 ± 5.1	2.9 ± 1.4	3.3 ± 1.1	18.6 ± 4.2
	MBP	25.5 ± 0.3	16.5 ± 1.4	16.5 ± 1.9	33.3 ± 3.4	95.3 ± 1.6	92.9 ± 2
	PDIb′a′	41.8 ± 10.8	33.3 ± 11	22 ± 3	36.2 ± 7.3	93.7 ± 2.7	95.1 ± 2.5
SHuffle T7	His	49.7 ± 2.9	38.8 ± 5.8	15 ± 2	5.8 ± 1.8	12.9 ± 0.9	62.5 ± 0.6
	MBP	24.1 ± 6.7	14.9 ± 1.6	6.3 ± 0.7	23.5 ± 4.6	61.6 ± 8.7	93.7 ± 3
	PDIb′a′	14.9 ± 3.6	18.2 ± 0.2	18 ± 1.8	28.3 ± 2.4	97 ± 1.1	97.1 ± 1.2

**Table 2:** Purification yield table from 0.5 L cultures.

Purification Step	Total Protein (mg)	Purity (%)	hGM-CSF (mg)	Yield (%)
Supernatant of whole *E. coli* lysate	118.8	23	27.3	100
1st IMAC	13.3	38	5.05	18.5
2nd IEC	0.7	98	0.68	13.5

## Data Availability

The datasets generated and analyzed during the present study are available from the corresponding author upon reasonable request.
